# The custom making of hierarchical micro/nanoscaled titanium phosphate coatings and their formation mechanism analysis†

**DOI:** 10.1039/c9ra08168b

**Published:** 2019-12-13

**Authors:** Bianyun Cai, Nan Jiang, Peijie Tan, Yi Hou, Yubao Li, Li Zhang, Songsong Zhu

**Affiliations:** Analytical & Testing Center, Sichuan University Chengdu 610065 China nic7504@scu.edu.cn nic1976@scu.edu.cn zhangli9111@126.com; State Key Laboratory of Oral Diseases, National Clinical Research Center for Oral Disease, West China Hospital of Stomatology, Sichuan University Chengdu 610065 China zss_1977@163.com

## Abstract

In this study, a series of hierarchical micro/nanoscaled titanium phosphate (TiP) coatings possessing various surface morphologies were successfully fabricated on titanium (Ti) discs. The hydrothermal reactions of Ti discs in hydrogen peroxide (H_2_O_2_) and phosphoric acid (H_3_PO_4_) mixed solution yield diverse topographies such as hemispheric clump, cylindrical rod, spherical walnut, micro/nano grass, micro/nano sheet, and fibrous network. And their crystal structures were mainly composed of Ti(HPO_4_)_2_·0.5H_2_O, (TiO)_2_P_2_O_7_, H_2_TiP_2_O_8_, Ti(HPO_4_)_2_ and TiO_2_. The morphology and crystal shape of the TiP coatings depend strongly on the mass ratio of H_2_O_2_/H_3_PO_4_, reaction temperature and water content. Besides, the formation mechanism of TiP coatings with diverse morphologies was explored from the perspective of energetics and crystallography. The mechanism exploration paved the way for custom-making TiP coatings with desirable micro/nanoscaled morphologies to meet specific application purposes. The *in vitro* cytological performances of TiP coatings were also evaluated by co-culturing with rat bone marrow stromal cells (BMSCs), demonstrating a positive prospect for their use in bone tissue engineering.

## Abstract 2

In this study, a series of hierarchical micro/nanoscaled titanium phosphate (TiP) coatings possessing various surface morphologies were successfully fabricated on titanium (Ti) discs.

## Graphical abstract

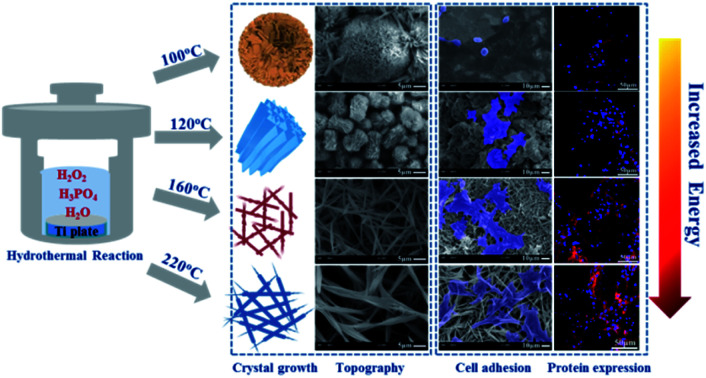

## 1. Introduction

Titanium (Ti)-based implants without surface modification generally lack initial osseointegration with surrounding bone tissues, leading to a short lifespan or even implantation failure.^1^ Therefore, various surface modifications including plasma-spraying,^2^ sandblasting,^3^ acid etching,^4^ alkali-heat treatment,^5^ anodic oxidation^6^ and surface coatings^7,8^ have been explored to modify the Ti surface structure, chemistry or topography while retaining the excellent bulk properties, among which, advanced surface coatings are the most widely applied methods to improve the osteoconductivity and osseointegration of Ti implants.

Bioinspired by the fact that natural bone possesses a micro/nano-scaled hierarchical structure mainly composed of calcium (Ca) and phosphorus (P), Sul *et al.*^9^ incorporated P into the flat titanium dioxide (TiO_2_) coating, and finally found increased bone-implant contact around the modified TiO_2_ coating. While Park *et al.*^10^ fabricated a crystalline P-incorporated Ti oxide surface, which exhibited enhanced MC3T4-E1 cells attachment, vitality and osteoblastic gene expressions. It has been analyzed that the enhanced biological performance of P-incorporated coatings stems from the truth that P-containing groups not only release a specific signal to induce osteoblasts differentiation,^11^ but also provide binding sites to attract Ca ions preferentially and form calcium phosphate, which is favorable to improve the bioactivity and biocompatibility of Ti implants.^12,13^ Recently, some efforts have focused on fabricating titanium phosphates (TiP) coatings with nanostructured morphology mimicking natural bone in both composition and microstructure through hydrothermal reactions of TiO_2_ powders or Ti plates in phosphate acid (H_3_PO_4_) solution.^14,15^ Especially, we have lately prepared TiP coatings with micro/nanoscaled hierarchical structure by reacting Ti substrates with hydrogen peroxide (H_2_O_2_)/H_3_PO_4_ mixed solution under hydrothermal conditions and also confirmed their greatly promoted osseointegration with surrounding bone tissues in comparison with the untreated Ti implants.^16^ Interestingly, we found the coating morphology is adjustable by regulating hydrothermal parameters, which triggers our great interest to explore the growth mechanism of TiP crystals with different morphologies.

According to the classical Curry–Wulff's rule, the crystal faces with the highest surface energy grows fastest to minimize the overall surface energy of all exposed crystal faces,^17^ which well explains the phenomenon that the same material often exhibits different crystal shapes resulting from the interference of environmental conditions.^18^ In our earlier work, it was found that in hydrothermal reactions, there were many variables (such as the mass ratio of H_2_O_2_/H_3_PO_4_, water content in H_2_O_2_/H_3_PO_4_ and reaction temperature) that influence the crystal growth, finally resulting in very different coating morphologies on Ti substrate.^16^ Therefore, making clear of how these variables affecting the crystal growth and shapes is of great significance for researchers to custom make desirable TiP crystals suitable for specific purposes.

It is well known that the crystallization process usually consists of nucleation and crystal growth.^19^ But only when the energy provided is enough to cross the potential barrier of nucleation can the critical crystal nuclei form,^20^ then followed by the crystal growth *via* ion-by-ion (or ion–molecules) attachment, which according to the Kossel's model of crystal layer growth, is a slow and complex process needing adequate energy.^21^ Therefore, we here hydrothermally fabricated TiP coatings on Ti discs with tailored crystal chemistry and surface topography by controlling the mass ratio of H_2_O_2_/H_3_PO_4_ (*R*), reaction temperature (*T*), and water content (*W*) in H_2_O_2_ and H_3_PO_4_ mixed solution. And the formation mechanism of these hierarchically structured TiP coatings as well as the correlation between the synthesis conditions and the formed crystal phase/morphology were explored from the perspective of energetics and crystallography. On this basis, cell responses to these TiP coatings formed on Ti discs were evaluated *in vitro* in order to provide strong evidence for TiP coated Ti implants to be used in orthopedic and dental applications.

## 2. Experimental section

### 2.1. Materials

Commercially pure titanium substrates (Ti, 99.99% purity, Baoji Titanium Industry Co. Ltd., China) were machined into *ϕ*10 mm × 1 mm discs. Hydrogen nitrate (HNO_3_), hydrogen fluoride (HF), acetone, ethyl alcohol, 30 wt% aqueous hydrogen peroxide (H_2_O_2_) and aqueous phosphoric acid (H_3_PO_4_, ≧85 wt% in H_2_O) were purchased from Chengdu Kelong Chemical Reagent Company. All chemicals were of analytical grade and used directly without further purification.

### 2.2. Synthesis of TiP coatings

After polished to mirror finish with 400, 800, 1200 and 2000 grit SiC sandpapers, sequentially, Ti discs were soaked in 10 mL of a mixture solution of HF : HNO_3_ : H_2_O at a volume ratio of 1 : 3 : 5 for 5 min to remove the naturally formed oxidized layer, then ultrasonically cleaned with acetone, alcohol and deionized water for 30 min, respectively. After that, Ti discs were placed into a mixed solution containing H_2_O_2_ and H_3_PO_4_ with different mass ratio and water content in a Teflon liner, which was then tightly sealed in an autoclave followed by hydrothermal treatment at different temperature varying from 80 °C to 220 °C for 24 h. Then, Ti discs were repeatedly washed under a strong stream of distilled water to remove the impurities on surfaces and finally dried in air at room temperature for 24 h.

### 2.3. Surface characterization

The surface and cross-sectional morphologies of TiP coatings were observed by scanning electron microscopy (SEM, JSM-6500LV, JEOL, Japan). X-ray diffraction (XRD, RINT-2000, Rigaku, Japan) was used to identify the phase compositions and the surface crystallinity of TiP coatings. The surface elemental compositions and mappings of TiP coatings were determined by energy-dispersive X-ray spectrometry (EDS, EPMA, JAX-8100, Japan). The chemical states of titanium (Ti), oxygen (O) and phosphorus (P) were analyzed by X-ray photoelectron spectroscopy (XPS, AXIS Ultra DLD, KRATOS, UK), outfitted with a monochromatic Al Kα radiation (*hν* = 1486.6 eV). The obtained data were analyzed by software of XPS Peak Fit 4.1. The contact angles of the samples against water were tested with Automatic Contact Angle Meter Model (SL200B, Solon Information Technology, China), and five random locations on each sample were measured to give the average value.

### 2.4. Cell isolation and culture

Cell isolation and culture were conducted in accordance with the Guidelines for Care and Use of Laboratory Animals of Sichuan University and experiments were approved by the Animal Research Committee of the State Key Laboratory of Oral Diseases and West China School of Stomatology, Sichuan University (approval number: SKLODLL2013A118). Briefly, bone marrow mesenchymal stem cells (BMSCs) were extracted from the femurs of 4 weeks old male Sprague-Dawley rats (Animal Research Center, Sichuan University, China) and cells at passage 2 were used for cell experiments.

### 2.5. Cell attachment assay

Ti discs were sterilized with Low Temperature Plasma Sterilizers (HRPS-120, Haier, Qingdao) for 2 h, and then soaked into PBS buffer solution for 24 h. BMSCs in the second passage were seeded onto the pre-wetted Ti disc (1 × 10^5^ cells per disc), and incubated in a humidified incubator at 37 °C with 5% CO_2_ for 4 h. Then, the sample was rinsed three times with PBS to remove those unattached cells. The remaining cells were fixed with 2.5% glutaraldehyde and were dehydrated in a graded series of ethanol solutions (20%, 40%, 60%, 80%, 90% and 100%). The morphology of cells attached to Ti disc was observed with SEM (JSM-6510LV, JEOL, Japan) after gold sputtering.

### 2.6. Cell immunofluorescence staining

Cell immunofluorescence staining and the expression of integrinβ1 and vinculin on the experimental Ti samples were detected by confocal laser scanning microscopy (CLSM). After incubation with Ti sample for 48 h, the cells were fixed in 4% paraformaldehyde and permeabilized with 0.2% Triton X-100. Nuclei were counterstained with DAPI and Ti samples were mounted on a glass slide. All procedures were completed in the dark, and all samples were observed using CLSM after thorough rinsing using PBS solution.

### 2.7. Cell proliferation assay

BMSCs were seeded on the sterilized Ti discs at a cell density of about 2 × 10^4^ cells mL^−1^. After co-culturing for 1, 3, 5 and 7 d, 20 μL of CCK-8 solution and 180 μL of culture medium were added to each well and then further incubated for 2 h. Finally, the absorbance of the incubated solution was measured by a microplate reader (Thermo, USA) at 450 nm wavelength.

### 2.8. ALP activity assay

The differentiation of BMSCs on sterilized Ti discs was evaluated by the ALP activity assay. BMSCs were seeded on Ti discs at a cell density of about 2 × 10^4^ cells per well and incubated for 7, 10 and 14 days, respectively. Then the cultured cells were fixed by 4% paraformaldehyde and ALP activity was determined at 405 nm using *p*-nitrophenyl phosphate as a substrate.

## 3. Results and discussion

### 3.1. Effects of water content in hydrothermal solution

**Fig. 1:**
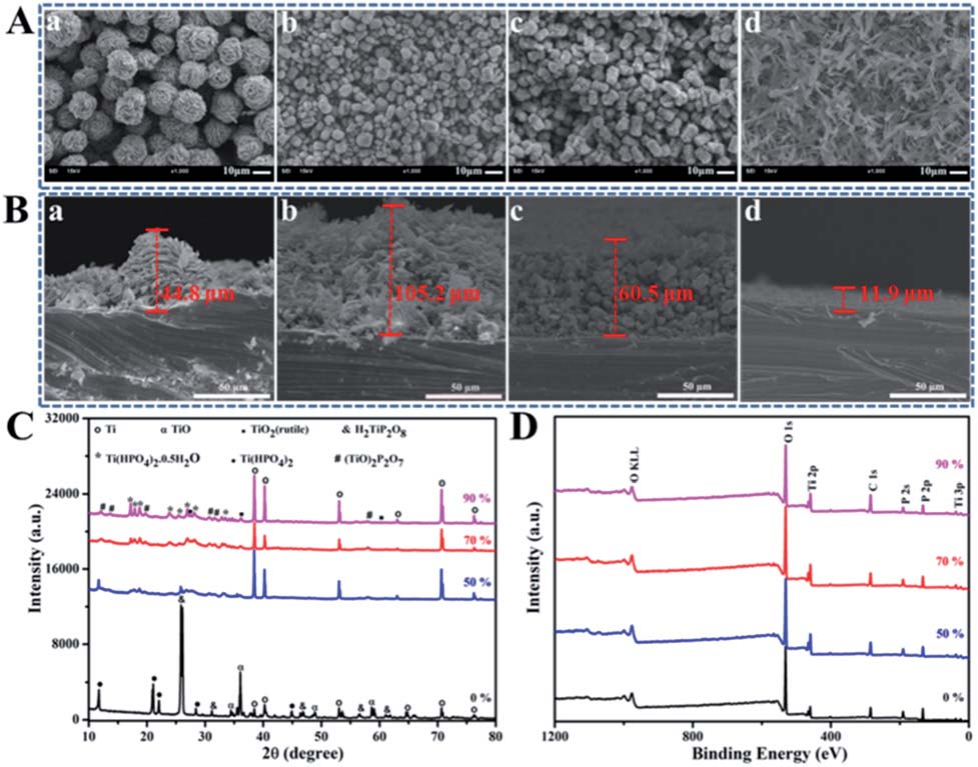
The SEM images showing the surface topography (A) and the cross-sectional morphology (B) of the TiP coatings reacted for 24 h in mixed solution with H_2_O_2_/H_3_PO_4_ at 9 : 1 under 120 °C with water content of 0 (a), 50% (b), 70% (c) and 90% (d); the XRD patterns (C) and XPS full spectra (D) of TiP coatings reacted for 24 h in mixed solution with H_2_O_2_/H_3_PO_4_ at 9 : 1 under 120 °C with water content changing from 0 to 90 wt%.

Hydrothermal method is based on dissolution reaction at the highly-stressed contact zones between particles in the presence of a solvent, which follows by a precipitation process on lower-stressed surfaces.^22^ As shown in SEM images, after hydrothermal treatment under 120 °C for 24 h in H_2_O_2_ and H_3_PO_4_ mixed solution, the surface morphologies of coatings formed on Ti discs evolved from spherical walnut-like, cylindrical rod-like to grass-like structures with water content changing from 0 to 90 wt% by setting *R* as the constant of 9 : 1 (Fig. 1A), and the corresponding coating thickness changed in the range of 11.9 to 105.2 μm (Fig. 1B). In a classically hydrothermal reaction, a solute dissolves and enters into a solution under certain temperature and pressure in form of ions or molecular groups, which are transported to the low temperature area (Ti discs) by convection generated by the temperature difference between the upper and lower parts of the reactor to form a supersaturated solution, then the nucleation occurs, followed by crystal growth. Therefore, more water evaporates to produce larger vapor and higher system pressure with the increase of water content in a closed reactor possessing the same volume.^23–25^ Meanwhile, higher system pressure can provide an additional driving force for the nucleation, urging TiP crystals to orientated grow along the crystal plane with higher energy, which helps explain why the coating structure transforms from spherical walnut-like structure (3 dimensional stable structure with low specific area) to cylindrical rod-like (2 dimensional) and even grass-like structures (1 dimensional) with water content increasing. XRD spectra shown in Fig. 1C confirm the crystalline phases formed on Ti discs in H_2_O_2_ and H_3_PO_4_ mixed solution with different water content. Specifically, the phases referring to Ti, TiO, H_2_TiP_2_O_8_ and Ti(HPO_4_)_2_ showed up without water, but with the increase of water content, Ti(HPO_4_)_2_ gradually transformed into Ti(HPO_4_)_2_·0.5H_2_O, while H_2_TiP_2_O_8_ further reacted with TiO_2_ to finally form (TiO)_2_P_2_O_7_. The XPS full spectra in Fig. 1D indicated that the formed coatings were mainly made up of Ti, O and P elements, whose high-resolution XPS spectra were exhibited in Fig. S1.† Obviously, the peaks of Ti_2p_ centered at 459.0 eV and 464.7 eV are assigned to the typical binding energies for Ti_2p_1/2__ and Ti_2p_3/2__ of Ti^4+^; the P_2p_ peak at 133.4 eV corresponds to the binding energy of P in PO_4_^3−^. In the O_1s_ spectra, the peaks at 530.32 eV and 531.6 eV are attributed to Ti–O–Ti in TiO_2_ and O in Ti–O–P and P=O, respectively, while the peak at 532.1 eV belongs to O in P–O–P.

### 3.2. Effects of mass ratio of H_2_O_2_/H_3_PO_4_ (*R*) in hydrothermal solution

Based on the effects of water content on the morphologies of TiP coatings, 70 wt% of water in solution was set for further study. The morphology evolution of TiP coatings after hydrothermally treated for 24 h under 120 °C in mixed solution with *R* varying from 1 : 9 to 9 : 1 (1 : 9, 1 : 7, 1 : 5, 1 : 3, 1 : 2, 1 : 1, 2 : 1, 5 : 1, 7 : 1 and 9 : 1, respectively) can be observed directly through SEM images shown in Fig. S2,† which indicates that with smaller *R* values (meaning less H_2_O_2_ in solution), the formed coatings mainly exhibited cylindrical rod-like structures (*R* varies from 1 : 9 to 1 : 1), then evolved to sheet-like and even grass-like structures along with enlarging *R* values (from 2 : 1 to 9 : 1). Accordingly, it can be analyzed that corrosion dominates the hydrothermal reaction when H_3_PO_4_ more than H_2_O_2_ is present in the mixed solution (*R* ≤ 1 : 1), together with plenty of Ti^4+^ ions continually releasing from Ti surface to supersaturate the solution, while H_2_O_2_ as an oxidizer can provide extra energy to accelerate nucleation process, then make TiP crystals grow into short rod-like structure (3 dimensional structure with low specific area); however, with the *R* value increasing to more than 1 : 1 till 9 : 1, high H_2_O_2_ content in the mixed solution together with high reaction pressure can offer enough energy to urge TiP crystals grow along the highest face and finally form sheet-like (2 dimensions) and even grass-like (1 dimension) coatings with higher specific area, indicating that H_2_O_2_ plays an important role for crystal shapes, which in turn result in the morphologic diversity of TiP coatings.

### 3.3. The effects of reaction temperature (*T*) in hydrothermal treatment

**Fig. 2:**
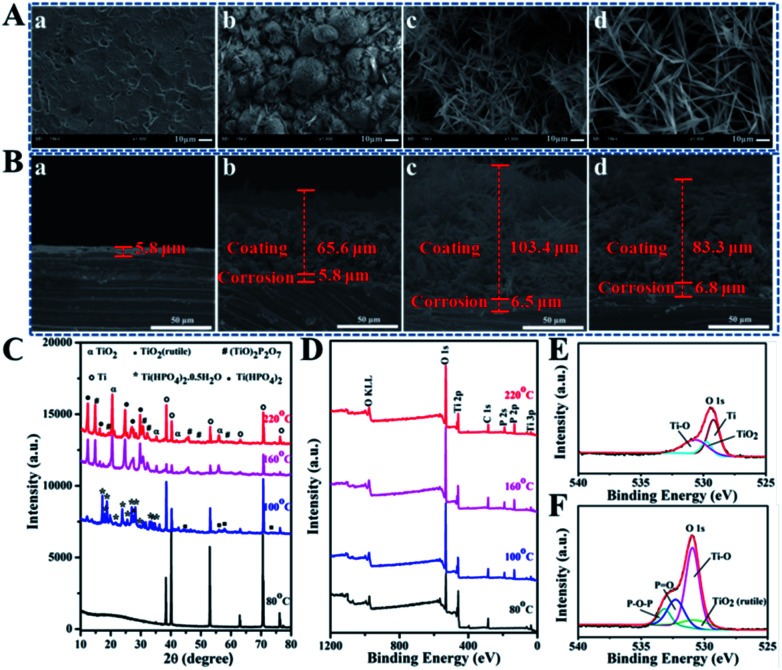
The SEM images showing the surface topography (A) and the cross-sectional morphology (B) of the TiP coatings reacted for 24 h in mixed solution with H_2_O_2_/H_3_PO_4_ at 9 : 1 containing 70 wt% water under 80 °C (a), 100 °C (b), 160 °C (c) and 220 °C (d); the XRD patterns (C) and XPS full spectra (D) of the TiP coatings reacted for 24 h in mixed solution with H_2_O_2_/H_3_PO_4_ at 9 : 1 containing 70 wt% water under 80 °C, 100 °C, 160 °C and 220 °C, respectively; the XPS high resolution spectra for O_1s_ of the TiP coatings prepared under 80 °C (E) and 220 °C (F).

According to the great contribution of H_2_O_2_ to the morphologic diversity of TiP coatings, we here set the *R* value as 1 : 2 to 9 : 1 for further experiments. SEM images in Fig. S3† detailedly reveal the morphologies of TiP coatings transform with varying *R* values (1 : 2, 1 : 1, 2 : 1, 5 : 1, 7 : 1, and 9 : 1) under different *T* (80, 100, 120, 140, 160, 180, 200, and 220 °C) after hydrothermally reacted for 24 h. Here, the temperature starting from 80 °C and ending with 220 °C is set because no coatings can grow on Ti discs in the hydrothermal system below 80 °C meanwhile the maximum temperature of Teflon liner can endure is 230 °C. Reacting under 80 °C for 24 h, Ti disc displays a relatively smooth surface with shallow pits in the range of *R* variations. While under 100 °C, the surfaces for all groups show hemispheric clumps-like structures consisted of numerous “petals” growing radially outward, but these hemispheres present a trend to grow bigger with *R* increasing. With the increase of *T*, the surface morphology undergoes the evolution from sheet-like to grass-like. As *T* is further increased, the coating exhibits network structure consisted of some randomly aligned slender fibers. In contrast, the influence factor of *T* has a much bigger impact on the surface topography of TiP coatings in comparison to *R*. Therefore, groups with *R* value at 9 : 1 and *T* of 80, 100, 160 and 220 °C were selected as representative candidates to further investigate on the structures and compositions (Fig. 2). When *T* is 80 °C, only a corrosion region composed of pure Ti about 5.8 μm thick is formed on the Ti substrate (Fig. 2A(a), B(a) and C). While the crystalline products referred to Ti, TiO_2(rutile)_ and Ti(HPO_4_)_2_·0.5H_2_O with hemispheric clumps-like structures and coating thickness around 65.6 μm except for a corrosion region about 5.8 μm thick grew on Ti disc under 100 °C (Fig. 2A(b), B(b) and C). With *T* increasing up to 160 °C, a reticular network structure where short and thin fibers are arranged randomly with about 103.4 μm coating thick and a corrosion region about 6.5 μm thick can be observed on Ti disc (Fig. 2A(c) and 2B(c)). And the XRD pattern marked as 160 °C in Fig. 2C shows that Ti(HPO_4_)_2_·0.5H_2_O transformed into Ti(HPO_4_)_2_ accompanied by the loss of a half crystal water together with the presence of a more stable compound of titanium oxide phosphate ((TiO)_2_P_2_O_7_) due to more dissolved Ti^4+^ reacting with abundant H_2_O_2_ and H_3_PO_4_. Keep improving *T* to 220 °C, the diffraction peaks assigned to Ti(HPO_4_)_2_ and (TiO)_2_P_2_O_7_ are markedly intensified (Fig. 2C) and the network coating showing a 83.3 μm thickness along with a 6.8 μm thick corrosion region is made up of much longer fibers (Fig. 2A(d) and 2B(d)).

The XPS full spectra in Fig. 2D show that the coatings prepared under 100, 160 and 220 °C are mainly composed of Ti, O and P elements, but the surface of Ti disc treated under 80 °C displays no trace of P but only Ti and O. In general, the atom losing an electron often generates a blue shift in the binding energy, on the contrary, red shift occurs. As shown in the high-resolution XPS spectra (Fig. S4†), about 1–2 eV blue shift in the electron binding energies for Ti_2p_1/2__ and Ti_2p_3/2__ on the surfaces of Ti discs can be seen with *T* going up from 80 °C to 220 °C because there are much more Ti atoms losing their electrons to be oxidized into Ti^4+^. The P_2p_ peaks at 133.4 eV correspond to the binding energy of P in PO_4_^3−^, suggesting the successful incorporation of P into the Ti surface. Moreover, the characteristic peaks attributed to Ti–O–P, P=O and P–O–P newly appear in the spectra of O_1s_ on the surfaces of samples hydrothermally treated under different *T* (Fig. S4† and 2F). The XPS data further testify that the newly formed TiP coatings are mainly composed of Ti(HPO_4_)_2_ and Ti(HPO_4_)_2_·0.5H_2_O, although not excluding the existence of free phosphate groups, which is consistent with the XRD results in Fig. 2C.

### 3.4. The wettability and P contents of Ti surfaces after hydrothermal treatment

**Fig. 3:**
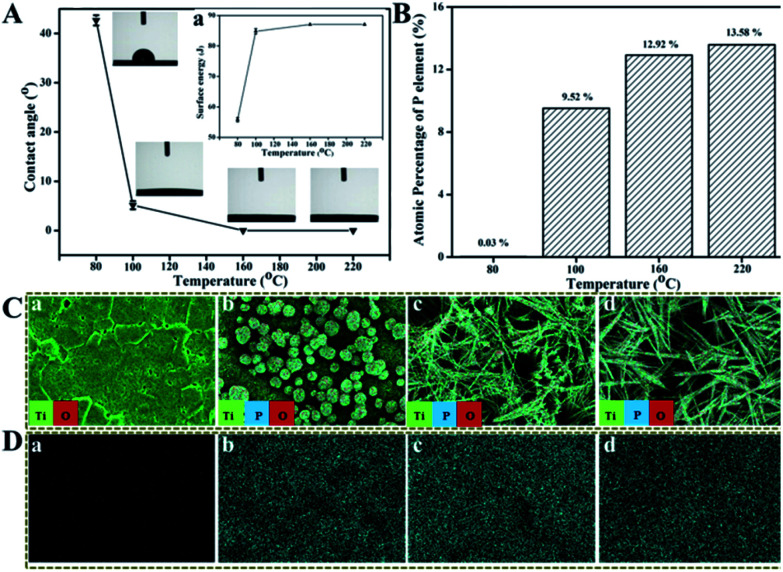
The water contact angle curves (A) and the corresponding surface energy curves (a) of TiP coatings reacted for 24 h in mixed solution with H_2_O_2_/H_3_PO_4_ at 9 : 1 containing 70 wt% water under 80 °C, 100 °C, 160 °C and 220 °C, respectively; the atomic percentage of P given by EDS (B), the merge images (C) and the distribution images of P (D) analyzed by EDS mapping of TiP coatings reacted for 24 h in mixed solution with H_2_O_2_/H_3_PO_4_ at 9 : 1 containing 70 wt% water under 80 °C (a), 100 °C (b), 160 °C (c) and 220 °C (d).

As reported,^26,27^ the surface wettability and surface free energy (SFE) of Ti implants have great impacts on the attachment of proteins or biomacromolecules which in turn influence the proliferation of osteoblasts and then the osteointegration of Ti implants. The hydrophilicity of these treated Ti surfaces were tested using contact angle (CA) measurement against water, through which the corresponding SFE was calculated, as shown in Fig. 3A. The water CA on Ti surface treated under 80 °C for 24 h is about 42.7°, and then decreases to about 5.6° under 100 °C whereas it decreases significantly to 0° with *T* increasing from 160 °C to 220 °C, implying that the TiP coatings formed above 160 °C behave super-hydrophilic property. The SFE is oppositely proportional to the CA of surfaces, that is, the smaller the CA, the higher the SFE (inset of Fig. 3A). The values of CA and SFE are listed in Table S1.†

The distribution and atomic percentage of P in TiP coatings formed on Ti discs under different *T* were characterized by EDS and surface mapping. It is obvious that the atomic percentage of P in coatings rises with *T* (Fig. 3B), the Ti surface treated under 80 °C mainly consists of Ti and O, as well as trace amount (0.03%) of P, but with *T* increasing, more and more P elements except for Ti and O are present in the formed coatings (Fig. 3C, D and S5†).

### 3.5. Formation mechanism of TiP coatings with diverse morphologies

In the hydrothermal system of this study, TiP coatings possessing diverse surface morphologies result from the reactions of Ti metal with H_2_O_2_ and H_3_PO_4_ in the solution, which involves a complex process including surface corrosion, oxidation, dissolution, nucleation, crystallization and re-deposition. Ti metal displays a dense hexagonal structure with lattice constants of *a* = 0.2911 nm and *c* = 0.46843 nm at room temperature. Under the help of H_3_PO_4_ in hydrothermal reaction, H_2_O_2_ are firstly decomposed into O_2_ to react with Ti atoms on the surface, thus generate lots of random defects (so-called “pitting attack”), resulting in a greatly enlarged surface area on Ti disc, enhanced surface free energy and chemical potential.^28,29^ With the increase of *T*, more H_2_O_2_, H^+^ and O_2_ are incorporated into the active Ti surface, facilitating the generation of Ti^4+^ as depicted in the following formula:^30^

1 2H_2_O_2_ → 2H_2_O + O_2_

2 Ti + 2H_2_O_2_ + 4H^+^ → Ti^4+^ + 4H_2_O

3 Ti + 4H^+^ → Ti^4+^ + H_2_

4 Ti + O_2_ + 2H_2_O → Ti^4+^ + 4OH^−^

With the pitting attack and the generation of Ti^4+^ continuing, more atomic vacancies generate, which enlarges the atomic spacing and expand the pore numbers at the corrosion region, and then increase the surface area and free energy of Ti samples, making the surface corrosion and oxidation reaction more intensive to form a loose surface on Ti substrate. Subsequently, more H_2_O_2_ molecules infiltrate into the loose surface on Ti substrate and react with those released Ti^4+^ to form different forms of the peroxohydroxo titanium complexes, such as cationic [Ti(O_2_)(OH)]^+^, [Ti(O_2_)(OH)_2_] and [Ti(O_2_)(OH)_3_]^−^.^31,32^ The peroxohydroxo titanium complexes are unstable and can be dimerized, leading to the formation of various oxoperoxohydroxo di-titanium complexes, such as [Ti_2_O_5_(OH)_2_], [Ti_2_O_5_(OH)_3_]^−^ and [Ti_2_O_5_(OH)_4_]^+^.^33^ At the same time, the complex of [Ti_2_O_5_(OH)_3_]^−^ are unstable and decompose into a precipitate of titanium hydroxide (Ti(OH)_4_).^34^ The corresponding reactions are described as follows.

5 3Ti^4+^ + 3H_2_O_2_ + 6H_2_O → [Ti(O_2_)(OH)]^+^ + [Ti(O_2_)(OH)_2_] + [Ti(O_2_)(OH)_3_]^−^ + 12H^+^

6 3[Ti(O_2_)(OH)_2_] + 3[Ti(O_2_)(OH)_3_]^−^ → [Ti_2_O_5_(OH)_2_] + [Ti_2_O_5_(OH)_3_]^−^ + [Ti_2_O_5_(OH)_4_]^+^ + 3H_2_O

7 [Ti_2_O_5_(OH)_3_]^−^ + H_2_O → Ti(OH)_4_ + 1/2O_2_ + OH^−^

Finally, TiP coatings with diverse morphologies deposit on Ti substrates accompanied by a series of physicochemical reactions between PO_4_^3−^, HPO_4_^2−^, H_2_PO_4_^−^ groups and Ti^4+^, H_2_O_2_ as well as the above-mentioned complexes probably in the following ways: (a) Ti(O_2_)(OH)_*n*−2_^(4−*n*)+^ or Ti_2_O_5_(OH)_*x*_^2−*x*^ continue to react with PO_4_^3−^, HPO_4_^2−^ and H_2_PO_4_^−^ groups in solution, leading to the deposition of amorphous or low crystallinity TiP coating; (b) high crystallinity TiP coating forms as the dissolved Ti-containing groups react with P-containing groups to organize into crystal clusters *via* the classical and/or non-classical pathways;^35^

8 Ti(OH)_4_ + 4H^+^ + 2HPO_4_^2−^ → Ti(HPO_4_)_2_ + 4H_2_O

(c) Ti^4+^ and H_2_O_2_ directly react with PO_4_^3−^, HPO_4_^2−^ and H_2_PO_4_^−^ groups to form polynuclear complexes with hierarchical structures and multi-components containing Ti, P, O, and H.

**Fig. 4:**
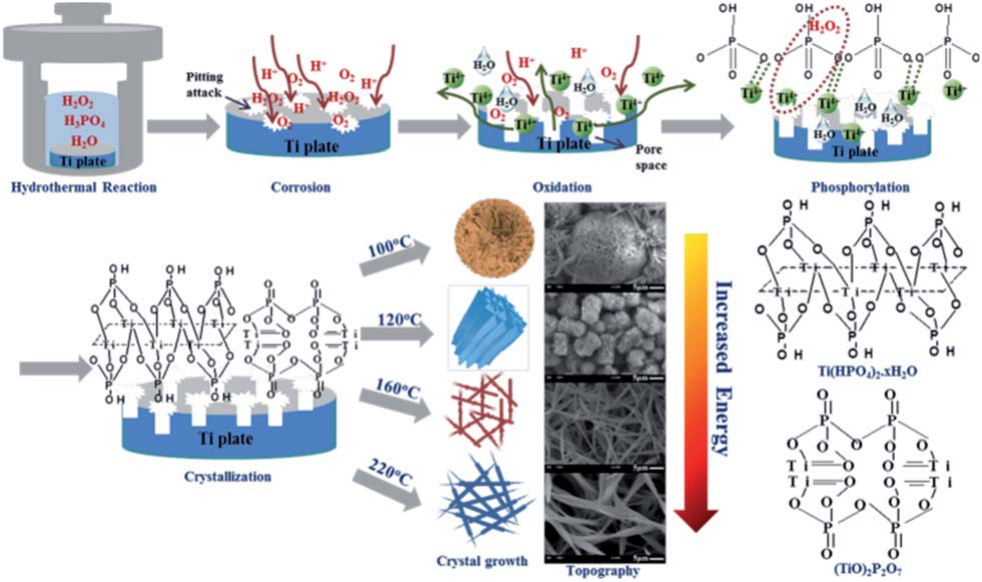
The schematic formation mechanism of the TiP coatings with different surface morphology formed on Ti discs.

It has been reported that only small amount of H_2_O_2_ decompose to generate O_2_ if heated under <100 °C;^36^ meanwhile, the set temperature of 80 °C is far below the boiling point of water (100 °C), not enough to vaporize water for promoting the reaction pressure to generate extra driving force. Both of the above reasons explain why no TiP coating except for a corrosion region with a ∼5.8 μm thickness formed on Ti substrate after hydrothermally treated for 24 h under 80 °C. With the temperature going up to 100 °C, which on one hand makes more H_2_O_2_ decompose into O_2_, on the other hand just equals to the boiling point of water, vaporizing part of water molecules to enhance the pressure in reactor, finally the energy accumulated in hydrothermal system is just enough to propel the formation of TiP coating with sphere-shaped morphology (3 dimensions) possessing low specific surface area in order to minimize the integrated surface energy,^37^ this process needs relatively less energy. Simultaneously, the decomposition of H_2_O_2_ is an exothermic reaction which generates a lot of heat further to provide energy for the hydrothermal system and also additional driving force for the nucleation of TiP crystals. Therefore, as the temperature and H_2_O_2_ content in system keep increasing (from 120 °C to 160 °C, then to 220 °C), abundant energy is provided to make TiP complexes grow faster in directions with higher surface energy, ultimately forming TiP coatings possessing cylindrical rod (2 dimensions) and fiber network (1 dimension) structures with a higher surface-to-volume ratio, as shown in Fig. 4.

### 3.6. Cytocompatibility of Ti samples with hydrothermal treatment

**Fig. 5:**
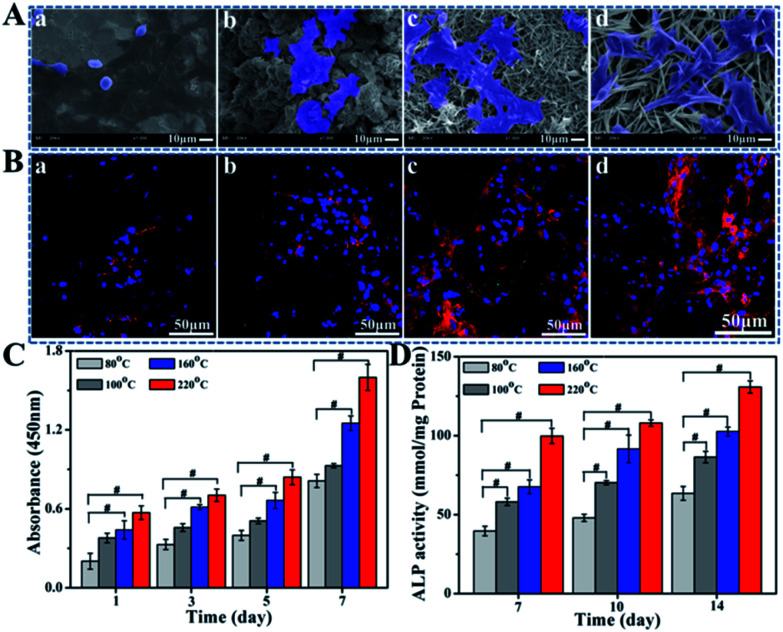
The BMSCs morphology investigated by SEM with 4 h incubation (A) and the integrin β1 and vinculin expressions of BMSCs investigated by CLSM after 48 h incubation (B) on the TiP coatings reacted for 24 h in mixed solution with H_2_O_2_/H_3_PO_4_ at 9 : 1 containing 70 wt% water under 80 °C (a), 100 °C (b), 160 °C (c) and 220 °C (d); the CCK-8 assay of the BMSCs cultured for 1, 3, 5, and 7 days (C) and the alkaline phosphatase activity (ALP) of BMSCs after culturing for 7, 10, and 14 days (D) on different Ti substrates. ^#^*p* < 0.05 compared to sample prepared under 80 °C.

As the precursor of osteoblasts, BMSCs play an important role in bone ecological stability and regeneration. The initial adhesion and spreading of BMSCs is the key factors that directly affect subsequent cellular functions and osseointegration.^38,39^ The adhesion status and morphologies of BMSCs on various implant surfaces after 4 h incubation are detected by SEM (Fig. 5A). For the sample hydrothermally processed under 80 °C, cells exhibit globular morphologies with limited spreading where few pseudopodia can be observed. In contrast, cells seeded on 100 °C or 160 °C hydrothermal groups spread much better, showing flat and stretching shapes. More fusiform or multilateral cells with more pseudopodia are observed on TiP coating with the increase of *T* to 220 °C, behaving their robust vitality.

The CLSM images exhibit the expression of integrinβ1 and vinculin. As shown in Fig. 5B and S6,† a stronger fluorescent intensity can be seen for fluorescence-stained cells cultured on TiP coatings prepared among *T* of 100, 160 and 220 °C compared with the group treated under 80 °C, which are in accordance with the SEM observation. To be specific, along with the increasing of *T*, more fluorescent signal can be clearly detected especially for samples prepared under 160 °C and 220 °C, suggesting that the formation of TiP coatings avail integrinβ1 and vinculin expressions, implying conducive cell adhesion.

CCK-8 assay was applied to measure the proliferation of BMSCs in order to investigate the biocompatibility of TiP samples. The results of the BMSCs cultured for 1, 3, 5, and 7 days are shown in Fig. 5C. The higher the processing temperature is, the better cell viability on these surfaces can be obtained. BMSCs on samples prepared under 80 °C show up the worst proliferating ability while they show the best on 220 °C group.

Cell differentiation is one of the key processes to evaluate the material biocompatibility. As a classical marker of differentiation of osteoblasts, ALP is detected in this study to confirm the cell mineralization.^40–42^ The ALP activity of BMSCs cultured on TiP coatings is shown in Fig. 5D. ALP activity of TiP coatings prepared under 100, 160 and 220 °C is notably greater than those prepared under 80 °C, indicating that the formation of TiP coatings was beneficial to cell osteogenic differentiation.

The better cell adhesion, proliferation and differentiation can be detected for TiP coatings prepared under 100 °C to 220 °C than the sample treated under 80 °C, which is mainly attributed to their excellent hydrophilicity, bigger surface free energy and higher atomic percentage of homo-dispersed P. On the other hand, BMSCs are extremely sensitive to acidic environment while TiP coatings are slightly acidic due to the residual of acid reactant or incomplete cleaning. Therefore, the improved cell responses for samples prepared under 160 °C and 220 °C are also attributed to the completely breaking down of the residual acid reactant accompanied by the increase of *T*.

## 4. Conclusion

A simple hydrothermal reaction of Ti discs in the mixed aqueous solutions of H_2_O_2_ and H_3_PO_4_ yielded various hierarchical micro/nanoscaled TiP coatings with a variety of crystal chemistry and surface topography by varying the mass ratio of H_2_O_2_/H_3_PO_4_, reaction temperature and water content of the hydrothermal solution. It is clarified that temperature, H_2_O_2_ and water content in the mixed solution play great roles in providing energy for the formation of TiP coatings with diverse morphologies. The formation mechanism of these TiP coatings was investigated comprehensively from the perspective of energetics and crystallography, providing conveniences for researchers to custom-make TiP coatings with desirable morphologies to meet specific purposes. The *in vitro* cytological performances of TiP coatings indicate that TiP coated Ti implants have the potential to be used in bone tissue regeneration.

## Conflicts of interest

There are no conflicts of interest to declare.

## Supplementary Material

RA-009-C9RA08168B-s001

## References

[cit1] Gellynck K., Shah R., Parkar M., Young A., Buxton P., Brett P. (2013). Bone.

[cit2] Becker W., Becker B. E., Ricci A., Bahat O., Rosenberg E., Rose L. F., Handelsman M., Israelson H. (2000). Clin. Implant Dent. Relat. Res..

[cit3] Rasmusson L., Kahnberg K. E., Tan A. (2001). Clin. Implant Dent. Relat. Res..

[cit4] Zinger O., Anselme K., Denzer A., Habersetzer P., Wieland M., Jeanfils J., Hardoui P., Landolt D. (2004). Biomaterials.

[cit5] Wu L. P., Qiu Y., Xi M., Li X. J., Cen C. P. (2015). New J. Chem..

[cit6] Sul Y. T., Johansson C. B., Roser K., Albrektsson T. (2002). Biomaterials.

[cit7] Barrere F., Valk C. M., Meijer G., Dalmeijer R. A. J., Groot K., Layrolle P. (2003). J. Biomed. Mater. Res..

[cit8] Chen X., Wang W. D., Cheng S., Dong B., Li Y. (2013). ACS Nano.

[cit9] Sul Y. T. (2003). Biomaterials.

[cit10] Park J. W., Kim Y. J., Jang J. H., Kwon T. G., Bae Y. C., Suh J. Y. (2010). Acta Biomater..

[cit11] Krupa D., Baszkiewicz J., Kozubowski J. A., Barcz A., Sobczak J. W., Bilinski A., Szumiel M. L., Rajchel B. (2001). Biomaterials.

[cit12] Krupa D., Baszkiewicz J., Kozubowski J. A., Barcz A., Sobczak J. W., Bilinski A., Szumiel M. L., Rajchel B. (2005). Biomaterials.

[cit13] Yang B. C., Uchida M., Kim H. M., Zhan X. D., Kokubo T. (2004). Biomaterials.

[cit14] Zhu Y., Hasegawa G., Kanamori K., Kiyomura T., Kurata H., Hayash K., Nakanishi K. (2017). CrystEngComm.

[cit15] Park J. W., Jang J. H., Lee C. S., Hanawa T. (2009). Acta Biomater..

[cit16] Jiang N., Guo Z. J., Sun D., Li Y. B., Yang Y. T., Chen C., Zhang L., Zhu S. S. (2018). ACS Nano.

[cit17] Privman V., Goia D. V., Park J. S., Matijevic E. (1999). J. Colloid Interface Sci..

[cit18] Malek J. (2000). Thermochim. Acta.

[cit19] Nanev C. N., Hodzhaoglu F. V., Dimitrov I. L. (2011). Cryst. Growth Des..

[cit20] Deij M. A., Los J. H., Meekes H., Vlieg E. (2006). J. Appl. Crystallogr..

[cit21] Gao H. J., Yang H., Wang S. F. (2016). Optik.

[cit22] Fang C. Q., Pu M. Y., Zhou X., Yang R., Lei W. Q., Wang C. X. (2018). J. Alloys Compd..

[cit23] Eriksson C., Nygren H. (2001). J. Lab. Clin. Med..

[cit24] Lai Y. K., Tang Y. X., Huang J. Y., Pan F., Chen Z., Zhang K. Q., Fuchs H., Chi L. F. (2013). Sci. Rep..

[cit25] Zhu Y., Hasegawa G., Kanamori K., Kiyomura T., Kurata H., Hayashi K., Nakanishi K. (2017). CrystEngComm.

[cit26] Thakral G. K., Thakral R., Sharma N., Seth J., Vashisht P. (2014). J. Clin. Diagn. Res..

[cit27] Jiang N., Du P. G., Qu W. D., Li L., Liu Z. H., Zhu S. S. (2016). Int. J. Nanomed..

[cit28] Wu J. M., Song X. M., Yan M. (2011). J. Hazard. Mater..

[cit29] Bavykin D. V., Kulak A. N., Walsh F. C. (2010). Cryst. Growth Des..

[cit30] Zhan H. Q., Yang X. F., Wang C. M., Liang C. L., Wu M. M. (2010). J. Phys. Chem..

[cit31] Zhang W. T., He F., Xie J. L., Liu X. Q., Fang D., Yang H., Luo Z. H. (2018). J. Non-Cryst. Solids.

[cit32] Kakihana M., Kobayashi M., Tomita K., Petrykin V. (2010). Bull. Chem. Soc. Jpn..

[cit33] Haider Z., Kang Y. S. (2014). ACS Appl. Mater. Interfaces.

[cit34] Mao Y. B., Kanungo M., Hemraj-Benny T., Wong S. S. (2006). J. Phys. Chem. B.

[cit35] An S. M., Su R., Hu Y. C., Liu J. B., Yang Y., Liu B. X., Guan P. F. (2018). Acta Mater..

[cit36] Chuan C. X. , Shi N. and Xu W., Forums of national petroleum and chemical production safety and control technology exchange, 2008, vol. 2, pp. 198–202

[cit37] Li H., Gong M., Yang A. P., Ma J., Li X. D., Yan Y. G. (2012). Int. J. Nanomed..

[cit38] Mitsunori Y., Yuko I., Ayako S., Toshio T., Takanori W. (2014). ACS Appl. Mater. Interfaces.

[cit39] Muhlebach J., Muller K., Schwarzenbach G. (1970). Inorg. Chem..

[cit40] Ignotz R. A., Massague J. (1987). Cell.

[cit41] Hoemann C. D., Gabalawy H. E., McKee M. D. (2009). Pathol. Biol..

[cit42] Li H., Gong M., Yang A. P., Ma J., Li X. D., Yan Y. G. (2012). Int. J. Nanomed..

