# Supplementary material for: The custom making of hierarchical micro/nanoscaled titanium phosphate coatings and their formation mechanism analysis
Source: RSC Adv. 2019 Dec 13;9(70):41311–8. doi: 10.1039/c9ra08168b (PMC9076429; doi:10.1039/c9ra08168b)
Supplement: RA-009-C9RA08168B-s001 [file RA-009-C9RA08168B-s001.pdf]

Electronic Supplementary Material (ESI) for RSC Advances.
This journal is © The Royal Society of Chemistry 2019

Supporting Information

The Custom Making of Hierarchical Micro/Nanoscaled Titanium Phosphate Coatings and Their formation Mechanism Analysis

Bianyun Cai[a], Nan Jiang[b], Peijie Tan[b], Yi Hou[a], Yubao Li[a]*, Li Zhang[a]*, Songsong Zhu[b]*

*The * marked authors are the corresponding authors*

[a] Analytical & Testing Center, Sichuan University, Chengdu 610065, China

[b] State Key Laboratory of Oral Diseases & National Clinical Research Center for Oral Disease & West China Hospital of Stomatology, Sichuan University, Chengdu 610065, China

E-mail: nic7504@scu.edu.cn (Y. Li);nic1976@scu.edu.cn, zhangli9111@126.com (L. Zhang); zss_1977@163.com (S. Zhu)

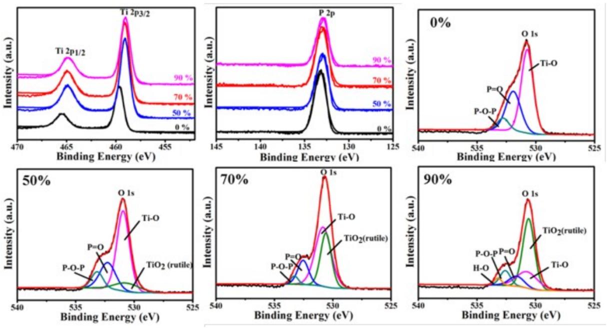

Fig. S1. The XPS high resolution spectra for Ti_{2p}, P_{2p} and O_{1s} of the TiP coatings reacted for 24 h in mixed solution with H_2O_2/H_3PO_4 at 9:1 under 120℃ with water content varying from 0 to 90%.

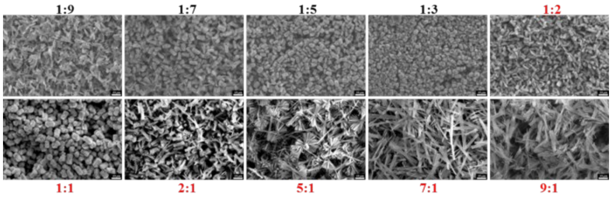

Fig. S2. SEM images showing the surface morphology of the TiP coatings reacted for 24 h in mixed solution with H_2O_2/H_3PO_4 varying from 1:9 to 9:1 containing 70wt% water under 120℃.

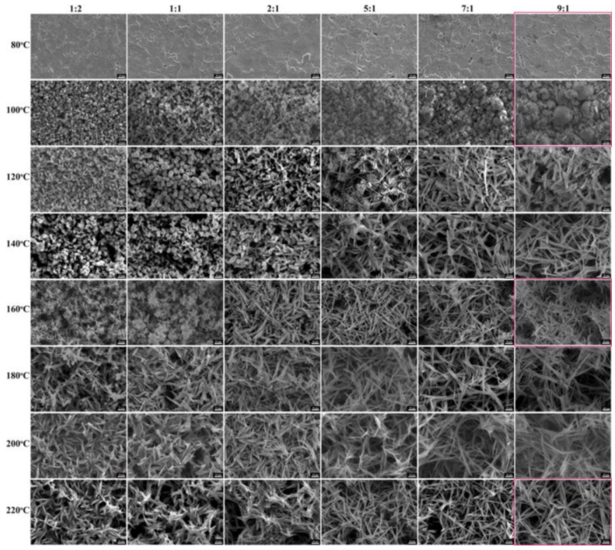

Fig. S3. SEM images showing the surface morphology of the TiP coatings reacted for 24 h in mixed solution with H_2O_2/H_3PO_4 varying from 1:2 to 9:1 containing 70wt% water under different temperature from 80℃ to 220℃.

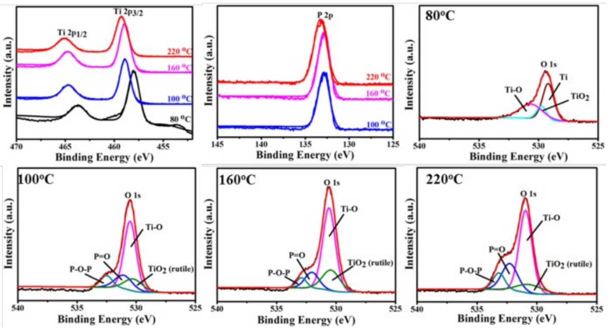

Fig. S4. The XPS high resolution spectra for Ti_{2p}, P_{2p} and O_{1s} of the TiP coatings reacted for 24 h in mixed solution with H_2O_2/H_3PO_4 at 9:1 containing 70wt% water under 80℃, 100℃, 160℃ and 220℃, respectively.

Table S1. The value of contact angle and surface energy for the TiP coatings reacted for 24 h in mixed solution with H_2O_2/H_3PO_4 at 9:1 containing 70wt% water under 80℃, 100℃, 160℃ and 220℃, respectively.

Sample	80℃	100℃	160℃	220℃
Contact Angle (^{o})	46.09±4.14	5.57±0.63	0	0
Surface Energy (J)	55.81±4.32	84.2±3.55	87.08	87.08

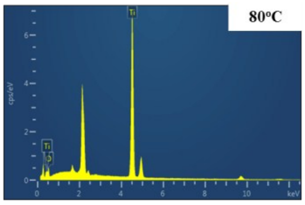

Element	Wt%	At%
O	11.48	27.98
Ti	88.52	72.02
Total	100.00	100.00

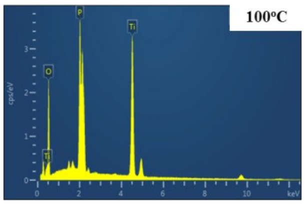

Element	Wt%	At%
O	40.93	65.2
P	11.57	9.52
Ti	47.50	25.28
Total	100.00	100.00

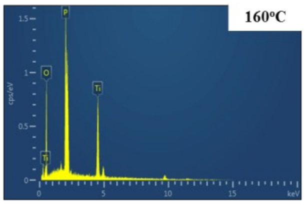

Element	Wt%	At%
O	45.51	68.95
P	16.87	12.92
Ti	36.62	18.13
Total	100.00	100.00

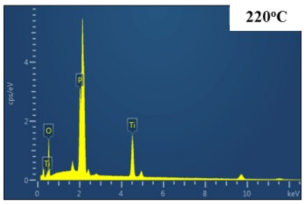

Element	Wt%	At%
O	45.25	69.00
P	17.19	13.58
Ti	37.56	17.42
Total	100.00	100.00

Fig. S5. The EDS analysis and element content of the TiP coatings reacted for 24 h in mixed solution with H_2O_2/H_3PO_4 at 9:1 containing 70wt% water under 80℃, 100℃, 160℃ and 220℃, respectively.

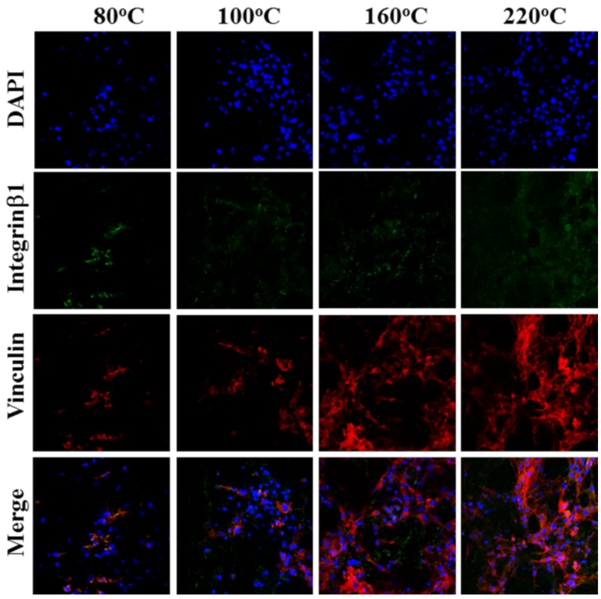

Fig. S6. The Integrinβ1 and vinculin expressions of BMSCs investigated by CLSM after 48 h incubation on the TiP coatings reacted for 24 h in mixed solution with H_2O_2/H_3PO_4 at 9:1 containing 70wt% water under 80℃, 100℃, 160℃ and 220℃, respectively.
